# Supplementary material for: Predictors of annual membership renewal to increase the sustainability of the Nepal National Health Insurance program: A cross-sectional survey
Source: PLOS Glob Public Health. 2022 Apr 4;2(4):e0000201. doi: 10.1371/journal.pgph.0000201 (PMC10021716; doi:10.1371/journal.pgph.0000201)
Supplement: S2 File — (DOCX) [file pgph.0000201.s002.docx]

**Questionnaire for the respondents who did not renew their members**

**Eligibility Check**

| Note to the interviewer:  All survey participants must meet the eligibility criteria. Responses to the eligibility screening questions are used to determine who is eligible to participate in the survey. This is the only segment that does not allow the participant to refuse answering a question. Any refusal to answer an eligibility criteria questions automatically makes the respondent ineligible.  Before asking specific questions to determine eligibility, all participants should be asked the first few moments to build rapport. | | | |
| --- | --- | --- | --- |
| **S.N.** | **Question** |  | **Response** |
| **1** | Were you the member of National Health Insurance (NHI) Program? | Yes……1  No…….2 | If the answer is no- the participant is not eligible to participate |
| **2** | Did you drop from the National Health Insurance (NHI) Program? | Yes……1  No…….2 | If the answer is no- the participant is not eligible to participate |
| **3** | Do you consent for the interview? | Yes……1  No…….2 | If the answer is no- the participant is not eligible to participate |
| *Note: The eligible respondent has to say NO to question 1&2 and provide consent for the study.* | | | |

### [ ] Check here to confirm that the inclusion criteria is met

### [ ] Check to confirm that the informed consent is obtained

1. Please give information sheet to the respondent and allow him/her to read it. Please read the information for the respondent if s/he is not able to read it.
2. If the respondent agrees to participate in the study "voluntarily", obtain his/her signature or thumb print (if the respondent is unable to write) in the form.
3. Signed consent is mandatory for this survey

### Identification tags

| 1 | Respondent code*: | \| [ ] [ ] \| [ ] [ ] \| [ ] [ ] \| \| --- \| --- \| --- \| \| District code \| Data collector code \| Respondent serial number \| |
| --- | --- | --- | --- | --- | --- | --- | --- | --- |
| 2 | Place of enrolment: | [ 1 ] Community [ 2 ] Health services center [ 3 ] Hospital [ 4 ] Self-help group  [ 5 ] Others, Please specify ……………………………………… |
| 3 | Name of the site, if applicable: |  |
| 4 | Location (City/Town/Village): |  |

### * District code: 061

*Shishir code: 00

* Ramu code: 99

### Interviewer information

| 1 | Interviewer's Name and signature: | .......................................................................  ....................................................................... |
| --- | --- | --- |
| 2 | Date of the interview | Day Month Year  - - |
| 3 | Interview start time | - |

**Part 1: Socio-demography characteristics**

| Q.N. | Questions | Answer/Code | Skip to |
| --- | --- | --- | --- |
| 1 | Age (complete the answer in years) | [ ] |  |
| 1.1 | Address of the participant  (City/Town/Village) |  |  |
| 1.2 | Do you have a poverty card | 1. [ ] Yes  2. [ ] No |  |
| 2 | Type of family- specify the number of family members | 1. [ ]Joint  2. [ ]Single |  |
| 2.1 | How many people currently live in your household, please also share how old are they? | 1. Total [ ]  2. Children (0-17) [ ]  3. Youth (18-24) [ ] 4. Adult (25-49) [ ]  5. Elderly (49 and above) [ ] |  |
| 3 | Do you consider yourself as male, female, transgender, or other? | 1. [ ] Male  2. [ ] Female  3. [ ] Transgender  4. [ ] Others (specify)………………..  5. [ ] Refused to answer |  |
| 4 | What is the level of your education? | 1. [ ] Never went to school  2. [ ] Never went to school but  can read and write Nepali language  3. [ ] Primary school  4. [ ] Secondary school  5. [ ] Higher secondary  6. [ ] College or university  7. [ ] I don’t know  8. [ ] Refused to answer |  |
| 5 | What is your occupation? | 1. [ ] Office employee (formal)  2. [ ] Self-employed (informal)  3. [ ] Employed, salaried, work for others (informal)  4. [ ] Unemployed  5. [ ] I don’t know  6. [ ] Refuse to answer  7. [ ] Other (Specify)…..…………… |  |
| 6 | How much is your monthly family income? | 1. [ ] ≤ 10,000 NRs  2. [ ] 10,001- 300, 00 NRs  3. [ ] 300,01- 50,000 NRs  4. [ ] 50,001-100,000 NRs  5. [ ] > 100,000 NRs  6. [ ] I don’t know  7. [ ] Refused to answer |  |
| 7 | What is your religion? | 1. [ ] Hinduism  2. [ ] Buddhism  3. [ ] Islam  4. [ ] Kirat  5. [ ] Christianity  6. [ ] I don’t know  7. [ ] Refused to answer  8. [ ]Other (specify)……………….. |  |

**Part 2: Reason for drop out**

*Note: The data collector can record this section after getting the consent. The record will be accessed only by the data collector and the principle researcher and it will be destroyed after the finalisation of the report.*

| Q.N. | Questions | Answer/Code | Skip to |
| --- | --- | --- | --- |
| 8 | When did your family join NHI? (Specify date and years) | [ ] [ ] |  |
| 8.1 | How much premium did you pay? (Specify the amount in NRs) | [ ] |  |
| 9 | How long where you a member of NHI? | 1. [ ] 1 year  2. [ ] 2 years  3. [ ] 3 years |  |
| 10 | Why did you decide not to renew your membership? | 1. [ ] No financial benefit perceived  2. [ ] Benefit package too little  3. [ ] Poor service and no medicine  4. [ ] Never utilized it  5. [ ] No money to renew  6. [ ] Others (please specify)……….. |  |
| 11 | If option 1, 2 or 3 selected- ask them to explain in detail |  | Open ended question. Data collector can record this answer. |
| 12 | If option 4 selected- explain them that their premium money will support the poorer family- after the end of the interview |  |  |
| 13 | If option 1 or 2  Did you make complain with your issues? | 1. [ ] Yes  2. [ ] No | If NO go to Q16 |
| 14 | If yes: Whom did they file or make your complain? | 1. [ ] Enrolment assistant  2. [ ] Enrolment officer  3. [ ] Province Manager  4. [ ] Hospital manager  5. [ ] Complain desk in Kathmandu Health Insurance Board office  6. [ ] Others (please specify)……… |  |
| 15 | Did they respond to your complain? | 1. [ ] Yes  2. [ ] No |  |
| 15.1 | If Yes- Was there any result or what happened after the complain was filed? |  | Open ended question |
| 16 | Why did you not file any complain? | 1. [ ] I did want not to  2. [ ] I did know where to  3. [ ] I did not believe in system  4. [ ] Others (please specify)…………. |  |
| 17 | What do you think should be done to improve the service? |  | Open ended question |
| 18 | Will you renew your membership if the service is improved? | 1. [ ] Yes  2. [ ] No  3. [ ] I don’t know | If No- please ask the reason |
