# Supplementary material for: Predictors of annual membership renewal to increase the sustainability of the Nepal National Health Insurance program: A cross-sectional survey
Source: PLOS Glob Public Health. 2022 Apr 4;2(4):e0000201. doi: 10.1371/journal.pgph.0000201 (PMC10021716; doi:10.1371/journal.pgph.0000201)
Supplement: S3 File — (DOCX) [file pgph.0000201.s003.docx]

**Survey questionnaire with the insured populations: Process evaluation**

**Eligibility Check**

| Note to the interviewer:  All survey participants must meet the eligibility criteria. Responses to the eligibility screening questions are used to determine who is eligible to participate in the survey. This is the only segment that does not allow the participant to refuse answering a question. Any refusal to answer an eligibility criteria questions automatically makes the respondent ineligible.  Before asking specific questions to determine eligibility, all participants should be asked the first few moments to build rapport. | | | |
| --- | --- | --- | --- |
| **S.N.** | **Question** | **Response** | **Remarks** |
| **1** | Are you the member of National Health Insurance (NHI) Program? | Yes……1  No…….2 | If the answer is no- the participant is not eligible to participate |
| **2** | Have you started your NHI benefit package? | Yes……1  No…….2 | If the answer is no- the participant is not eligible to participate |
| **2.1** | How long has it been since your benefit package has started?  Mention in months or year | ………………………… | If less than 9 months stop the interview, the participant is not eligible |
| **3** | Have you or any members of your household utilized the NHI benefit package at least one once you became NHI members? | Yes……1  No…….2 | If the answer is no- the participant is not eligible to participate |
| **4** | Do you consent for the interview? | Yes……1  No…….2 | If the answer is no- the participant is not eligible to participate |
| *Note: If any respondent fulfills question 2&2.1 and confirm yes (Y) for both questions 3&4 please proceed ahead with the full interview- otherwise look for next respondents.* | | | |

### [ ] Check here to confirm that the inclusion criteria is met

### [ ] Check to confirm that the informed consent is obtained

- Please give information sheet to the respondent and allow him/her to read it. Please read the information for the respondent if s/he is not able to read it.
- If the respondent agrees to participate in the study "voluntarily", obtain his/her signature or thumb print (if the respondent is unable to write) in the form.
- Signed consent is mandatory for this survey

### Identification tags

| 1 | Respondent code*: | \| [ ] [ ] \| [ ] [ ] \| [ ] [ ] \| \| --- \| --- \| --- \| \| District code \| Data collector code \| Respondent serial number \| |
| --- | --- | --- | --- | --- | --- | --- | --- | --- |
| 2 | Place of enrolment: | [ 1 ] Community [ 2 ] Health services center [ 3 ] Hospital [ 4 ] Self-help group  [ 5 ] Others, Please specify ……………………………………… |
| 3 | Name of the site, if applicable: |  |
| 4 | Location (City/Town/Village): |  |

### * District code: 061

*Shishir code: 00

* Ramu code: 99

### Interviewer information

| 1 | Interviewer's Name and signature: | .......................................................................  ....................................................................... |
| --- | --- | --- |
| 2 | Date of the interview | Day Month Year  - - |
| 3 | Interview start time | - |

**Part 1: Socio-demography characteristics**

| Q.N. | Questions | Answer/Code | Skip to |
| --- | --- | --- | --- |
| 1 | Age (complete the answer in years) | [ ] |  |
| 1.1 | Address of the participant  (City/Town/Village) |  |  |
| 1.2 | Do you have a poverty card | 1. [ ] Yes  2. [ ] No |  |
| 2 | Type of family- specify the number of family members | 1. [ ]Joint  2. [ ]Single |  |
| 2.1 | How many people currently live in your household, please also share how old are they? | 1. Total [ ]  2. Children (0-17) [ ]  3. Youth (18-24) [ ] 4. Adult (25-49) [ ]  5. Elderly (49 and above) [ ] |  |
| 3 | Do you consider yourself as male, female, transgender, or other? | 1. [ ] Male  2. [ ] Female  3. [ ] Transgender  4. [ ] Others (specify)………………..  5. [ ] Refused to answer |  |
| 4 | What is the level of your education? | 1. [ ] Never went to school  2. [ ] Never went to school but  can read and write Nepali language  3. [ ] Primary school  4. [ ] Secondary school  5. [ ] Higher secondary  6. [ ] College or university  7. [ ] I don’t know  8. [ ] Refused to answer |  |
| 5 | What is your occupation? | 1. [ ] Office employee (formal)  2. [ ] Self-employed, Business (informal)  3. [ ] Employed, salaried, work for others (informal)  4. [ ] Unemployed  5. [ ] I don’t know  6. [ ] Refuse to answer  7. [ ] Other (Specify)…..…………… |  |
| 6 | How much is your monthly family income? | 1. [ ] ≤ 10,000 NRs  2. [ ] 10,001- 300, 00 NRs  3. [ ] 300,01- 50,000 NRs  4. [ ] 50,001-100,000 NRs  5. [ ] > 100,000 NRs  6. [ ] I don’t know  7. [ ] Refused to answer |  |
| 7 | What is your religion? | 1. [ ] Hinduism  2. [ ] Buddhism  3. [ ] Islam  4. [ ] Kirat  5. [ ] Christianity  6. [ ] I don’t know  7. [ ] Refused to answer  8. [ ]Other (specify)……………….. |  |

**Part 2: Becoming a NHI member**

| Q.N. | Questions | Answer/Code | Skip to |
| --- | --- | --- | --- |
| 8 | When did your family join NHI? (Specify date and years) | [ ] [ ] |  |
| 8.1 | How much premium did you pay? (Specify the amount in NRs) | [ ] |  |
| 8.2 | Do you think the premium is affordable and reasonable for you? | 1. [ ] Yes  2. [ ] No |  |
| 8.3 | How did you pay? | 1. [ ] Did not have to pay  2. [ ] Usual salary, pensions and regular household income  3. [ ] Worked over time  4. [ ] Sold jewellary/belongings  5. [ ] Sold property  6. [ ] Borrowed money from the money lender  7. [ ] Borrowed money from friends/relatives  8. [ ] Borrowed money from employers  9. [ ] Borrowed money from bank  10. [ ] Used the savings  11. [ ] Used Investment fund  12. [ ] Remittance  13. [ ] Others (specify)……………… |  |
| 9 | How did you know about the NHI? | 1. [ ] Enrollment assistant  2. [ ] Newspaper/tv/radio advertisement  3. [ ] Community gathering  4. [ ] NHI awareness program  5. [ ] Friends/relatives who were  already insured  6. [ ] Others (specify)……………… |  |
| 10 | Do you think the first information you received about the NHI was clear to you? | 1. [ ] Yes  2. [ ] No  3. [ ] Don’t know | If Yes please go to question 11  *SQ |
| 10.1 | If no- what was not clear | 1. [ ] Enrolment process  2. [ ] Benefit package  3. [ ] Importance of NHI  4. [ ] Others (specify)……………… |  |
| 10.2 | Where did you get the clear information? | 1. [ ] Enrollment assistant  2. [ ] NHI Office  3. [ ] Current NHI members  4. [ ] Others (specify)…………….. |  |
| 11 | How long was the enrollment process? | 1. [ ] Less than 1 week  2. [ ] 1 week  3. [ ] 2 weeks  4. [ ] 3 weeks  5. [ ] 4 weeks  6. [ ] More than 1 month | *SQ |
| 12 | If more than 1 month, what was the reason? | 1. [ ] Slow process from enrollment  Assistant  2. [ ] Digital system malfunction  3. [ ] Enrollment officer/province  manager away f or long time  4. [ ] Could not pay the premium  on time  5. [ ] Others (specify)………………. |  |
| 13 | How do you describe the overall process of you becoming a member? | 1. [ ] Easy  2. [ ] Difficult  3. [ ] Don’t know | *SQ |
| 14 | Did the scheme (service package) start in time after you became the NHI member? | 1. [ ] Yes  2. [ ] No  3. [ ] Don’t know | Max two months after the membership  *SQ |
| 15 | If no why? | 1. [ ] Digital system malfunction  2. [ ] Enrollment officer/province  manager away for long time  3. [ ] The service provider not  ready to provide service  4. [ ] Others (specify)………………. |  |

**Part 3: Performance of enrollment assistants and officers**

| Q.N. | Questions | Coding Categories | Skip to |
| --- | --- | --- | --- |
| 16 | Do you know how many enrollment assistants are there in your ward? | 1. [ ] Yes  2. [ ] No |  |
| 16.1 | Specify the number of enrollment assistant you know | [ ] |  |
| 16.2 | Do you think the number of enrollment assistant is enough in your ward? | 1. [ ] Yes  2. [ ] No  3. [ ] Don’t know |  |
| 16.3 | Are they able to reach out to all the households in your ward? | 1. [ ] Yes  2. [ ] No  3. [ ] Don’t know |  |
| 17 | Did you go to the enrolment assistant or did enrolment assistant reach out to you? | 1. [ ] I went to the enrolment assistant  2. [ ] Enrolment assistants visited us |  |
| 18 | How many times did they visit your house until you became the members? | 1. [ ] 1-3 times  2. [ ] 4-6 times  3. [ ] 7-9 time  4. [ ] More than 10 times  5. [ ] Don’t know |  |
| 19 | What did they do when they visit you? | 1. [ ] Explained about NHI  2. [ ] Motivated you to be  NHI member  3. [ ] Gossiped about NHI, Enrollment officer  4. [ ] Shared their frustrations  5. [ ] Provided you with IEC on NHI  6. [ ] Others (specify)……………. | Multiple choice |
| 20 | Did the enrolment assistant provide the clear and accurate information about NHI? | 1. [ ] Yes  2. [ ] No  3. [ ] Don’t know | If Yes please go to question 22  *SQ |
| 21 | If no, how did you get the clear information? | 1. [ ] Went to NHI district office  2. [ ] Asked the enrolment assistant  again  3. [ ] Asked the current NHI members  4. [ ] Others (specify)……………… |  |

**Part 4: Benefit packages**

| Q.N. | Questions | Coding Categories | Skip to |
| --- | --- | --- | --- |
| 22 | Do you know the amount of benefit package you receive of becoming NHI? | 1. [ ] Yes  2. [ ] No |  |
| 22.1 | How much is the amount per year? (specify in NRs) | [ ] |  |
| 23 | Which health facility did you chose as your first point of contact? | 1. [ ] PHC  2. [ ] Public Hospital  3. [ ] Private Hospital  4. [ ] Others (specify)…………….. |  |
| 24 | Did you have enough options to chose the point of contact? | 1. [ ] Yes  2. [ ] No  3. [ ] Don’t know | *SQ |
| 25 | How did you choose the first point of service center? | 1. [ ] Chose on my will  2. [ ] Enrollment assistant asked to choose  3. [ ] No choices to choose my preference  4. [ ] Others (specify)…………….. |  |
| 25.1 | Please provide name of your first contact of PHC or hospital. |  |  |
| 26 | Why did you choose PHC/hospital as the first point of contact? | 1. [ ] Near to my place  2. [ ] Been their many times (comfortable)  3. [ ] Quality services  4. [ ] Friendly health workers  5. [ ] More facilities  6. [ ] Others (specify)……………… | Multiple selection |
| 27 | How many times did you visit your PHC/hospital, in last 9 months, after becoming the NHI members? | 1. [ ] 1-3 times  2. [ ] 4-6 times  3. [ ] 7-9 time  4. [ ] More than 10 times |  |
| 28 | Did you visit the PHC/hospital more often after becoming the NHI members? | 1. [ ] Yes  2. [ ] No  3. [ ] Don’t know |  |
| 28.1 | If yes- why? | 1. [ ] Free treatment  2. [ ] Free medicine  3. [ ] Others (specify)…………… |  |
| 29 | How was the procedure of getting the service after becoming NHI member? | 1. [ ] Easy- more priority given  2. [ ] Lengthy process with forms  and formalities (long waiting hours  3. [ ] Difficult- lesser priority given  4. [ ] No changes  5. [ ] Others (specify | *SQ |
| 30 | Did you find any changes in the PHC/hospital services, facilities and environment after becoming NHI members? | 1. [ ] Yes  2. [ ] No  3. [ ] Don’t know | If no- go to 31 |
| 30.1 | If yes- what are the changes? | 1. [ ] Addition of facilities  2. [ ] Addition of services  3. [ ] Separate system for NHI members  4. [ ] Increased number of patients  5. [ ] Others (specify)……………. | *SQ |
| 31 | Did you find any changes in the behaviors and attitude of health service providers after becoming NHI members? | 1. [ ] Yes  2. [ ] No  3. [ ] Don’t know | If no- go to 32 |
| 31.1 | If yes- please specify | 1. [ ] More attention to NHI members  2. [ ] Less attention to NHI members  3. [ ] Others (specify)……………. | *SQ |
| 32 | How much of benefit package did you use after becoming NHI member in first year? | 1. [ ] None  2. [ ] 1- 25%  3. [ ] 26-50%  4. [ ] 51-75%  5. [ ] 76-99%  6. [ ] 100% | If 100% utilized please go to 33 |
| 32.1 | If chosen 1 or 2- please ask the reason | 1. [ ] Nobody fell sick seriously  2. [ ] Did not use the selected PHC/hospital  3. [ ] Visited private hospital  4. [ ] Others (specify)………………. |  |
| 33 | If 100% chosen, how long did you take to use 100% | 1. [ ] 1-3 months  2. [ ] 4-6 months  3. [ ] 7-10 months  4. [ ] 11-12 months |  |
| 34 | How did you manage the expense after completing 100%? | 1. [ ] Did not visit the center  2. [ ] Out of pocket expense  3. [ ] Other insurance  4. [ ] Free health services  5. [ ] Others……………………… |  |
| 35 | Was the information about the status of your benefit package easily accessible? | 1. [ ] Yes  2. [ ] No  3. [ ] Don’t know | *SQ |
| 36 | Were you referred to other health facilities from your PHC/hospital? | 1. [ ] Yes  2. [ ] No  3. [ ] Don’t know | If No go to 39 |
| 37 | How many times you were referred in last three months? | 1. [ ] 1-3 times  2. [ ] 4-6 times  3. [ ] 7-9 time  4. [ ] More than 10 times |  |
| 38 | How was the referral mechanism? | 1. [ ] Easy- more priority given  2. [ ] Lengthy process with forms  And formalities  3. [ ] Difficult- lesser priority given  4. [ ] Others (specify).……………… | *SQ |
| 39 | Are the drugs available all the time in your PHC/hospital? | 1. [ ] Yes  2. [ ] No  3. [ ] Don’t know | If 1- then go to 40 |
| 39.1 | How many times did you not get the drugs from your PHC/hospital in last 3 months? | 1. [ ] 1-3 times  2. [ ] 4-6 times  3. [ ] 7-9 time  4. [ ] More than 10 times |  |
| 39.2 | What did you do? | 1. [ ] Did not buy  2. [ ] Bought from private pharmacy  3. [ ] Bought from other PHC and hospital | If 1- then go to 40 |
| 39.3 | If 2 or 3: how did you buy it? | 1. [ ] Out of pocket  2. [ ] Reimbursed  3. [ ] Others (specify)……………… |  |
| 40 | Are you satisfied with the your service provider? | 1. [ ] Yes  2. [ ] No  3. [ ] Don’t know | If no- go to 40.2 |
| 40.1 | If yes, what are the reasons? | 1. [ ] Free quality services  2. [ ] More emphasis for NHI member 3. [ ] Free drugs  4. [ ] Others (specify).…………… |  |
| 40.2 | If not, what are the reasons? | 1. [ ] Long waiting hours than before  2. [ ] Drugs not available frequently  3. [ ] Lesser emphasis for NHI member  4. [ ] Others (specify).…………….. |  |
| 41 | Did you ever filed a complain when you are not satisfied with the service providers? | 1. [ ] Yes  2. [ ] No  3. [ ] Refused to answer | If No- go to 41.2 |
| 41.1 | If yes, Where did you file the complain? | 1. [ ] Hospital management  2. [ ] NHI district office  3. [ ] NHI head office in Kathmandu  4. [ ] Others (specify).……………… |  |
| 41.2 | If no, do you know where to file the complain? | 1. [ ] Yes  2. [ ] No  3. [ ] Don’t know |  |

**Part 5: Replacement of lost card and renewal**

| Q.N. | Questions | Coding Categories | Skip to |
| --- | --- | --- | --- |
| 42 | Have you ever lost the card? | 1. [ ] Yes  2. [ ] No | If NO go- 44 |
| 43 | How long did it take to replace the card? | 1. [ ] Less than 1 week  2. [ ] 1 week  3. [ ] 2 weeks  4. [ ] 3 weeks  5. [ ] 4 weeks  6. [ ] More than 1 month |  |
| 43.1 | If more than 2 weeks- why? | 1. [ ] Slow process from enrollment  Assistant  2. [ ] Digital system malfunction  3. [ ] Enrollment officer/district  manager away for long time  4. [ ] Discrepancy in the name  5. [ ] Others (specify)………………. |  |
| 44 | How many times did you renew your membership? | 1. [ ] 0 time  2. [ ] 1 time  3. [ ] 2 times  4. [ ] 3 times | If 0 time go to 48 |
| 45 | Who reminded you to renew the membership? | 1. [ ] Myself  2. [ ] Enrolment assistant  3. [ ] Mobile SMS from NHI  4. [ ] Others (specify)……………… |  |
| 46 | Who supported you with the renewal process? | 1. [ ] Enrolment assistant  2. [ ] Enrolment officer  3. [ ] Others (specify)……………… |  |
| 47 | How was the process of renewal? | 1. [ ] Easy  2. [ ] Difficult  3. [ ] Don’t know | *SQ |
| 48 | Will you renew again? | 1. [ ] Yes  2. [ ] No  3. [ ] Don’t know | If NO or I don’t know go to 48.2 |
| 48.1 | If yes- why did you decide to renew the membership? | 1. [ ] Reduced by out of pocket expense  2. [ ] Protected my family from  Catastrophic expense  3. [ ] Improved the health of my family  4. [ ] Better services and processes  5. [ ] Better benefit package  6. [ ] Others (please specify)………… | Multiple choice  *SQ |
| 48.2 | If NO or I don’t know- why | 1. [ ] No financial benefit perceived  2. [ ] Benefit package too little  3. [ ] Poor service and no medicine  4. [ ] Never utilized it  5. [ ] No money to renew  6. [ ] Others (please specify)……….. |  |
| 49 | Any specific recommendations to make the program and service better to meet your needs.  2-3 specific recommendations |  |  |

***SQ: Satisfactory questions**
