# Supplementary material for: Predictors of annual membership renewal to increase the sustainability of the Nepal National Health Insurance program: A cross-sectional survey
Source: PLOS Glob Public Health. 2022 Apr 4;2(4):e0000201. doi: 10.1371/journal.pgph.0000201 (PMC10021716; doi:10.1371/journal.pgph.0000201)
Supplement: Attachment — Submitted filename: Response to reviewers-GG.docx [file pgph.0000201.s006.docx]

PGPH-D-21-01035
Predictors of annual membership renewal to increase the sustainability of the Nepal National Health Insurance program: a cross-sectional survey
PLOS Global Public Health

Response to the reviewers and editors comments

| Reviewers comments | Authors response |
| --- | --- |
| **Reviewer # 1** | |
| The manuscript presents an investigation of Predictors of annual membership renewal to increase the sustainability of the Nepal National Health Insurance program among informal sectors using mixed methods. The manuscript is generally well written and presented. | The authors extend thanks to reviewer #1 for pointing out the strengths of the manuscript. |
| **Reviewer # 2** | |
| **R2Comment 1:** The title sounds good and reflects the context of the paper. The author gave all necessary background information but can also be improved upon. The methodology approach is good. Sample size computation and sampling technique is scientifically grounded. | The authors extend thanks to reviewer #2 for highlighting the strengths of the introduction and methodology. |
| **R2Comment 2:** However, the analysis carried out and the conclusion should be checked. | The authors have revised the data analysis as mentioned under R2Comment 3 response. In summary, the chi-square test has been replaced with univariate logistic regression to obtain unadjusted OR (Odd Ratio) and the collinearity test has been conducted employing VIF (Variation Inflation Factor) value only.  The authors are not quite sure what particular content should be checked in the conclusion. The abstract section conclusion has been revised to strengthen the content. |
| **R2Comment 3:** The author mentioned chi-square to test relationship while simple correlation Pearson will establish relationships between the independent variable and dependent variables. | The authors have replaced chi-square with univariate logistic regression given that P-value from both processes would be similar. In particular, upon further discussion with the statistical expert, it was advised to calculate unadjusted OR to be compared with adjusted OR, which would be only possible from univariate logistic regression.  Similarly, Pearson correlation was employed to check the collinearity after selecting variables to include in multivariable analysis, together with the VIF (Variable Inflation Factor). Since Pearson co-relation could be misleading, we omitted it from the data analysis and only retained the VIF value for clarity. |
| **R2Comment 4**: Predicting variables were clearly stated connecting with title. For instance, three factors such as income, health quality and health service utilization were stated as predicting factors for membership renewal of health insurance. | The authors extend thanks for pointing out the clear connection of the title with the identified predicting variables. The study objective has been revised to make the connections more clear as follows   - Determine the predictors of intention to renew annual subscription for NHI program among enrolled members.   We have also highlighted key predictors at the very beginning of the discussion and subsequently provided further analysis to them. |
| **Reviewer #3** | |
| **R3Comment 1:** The authors have chosen a very interesting and relevant topic for Nepal. However, the structure of paper is not in PLOS GLOBAL Health format and writing style is very poor. | The authors thank reviewer 3 for highlighting the strengths and weaknesses of the manuscript. The revisions made to fully meet the PLOS Global Health Format are as follows.   - Abstract with all essential and adequate information, which includes objectives, additional information to method and results, and conclusion. - The tables are re-worked to be reader-friendly. - English language editing and revisions have now been made to the entire manuscript by a UK-based native English-speaking linguistic editor and member of the CIEP (Chartered Institute of Editing and Proofreading, UK). The paper uses American spelling throughout and has been checked for consistency in this respect. The linguistic editor can be reached via [marinamoorechula@gmail.com](mailto:marinamoorechula@gmail.com). |
| **R3Comment 2:** The abstract has not mentioned objective of the study as well as conclusion of the study. | The authors have included study objectives in the revised abstract. The conclusion content was included in the discussion of the abstract. The revised abstract has replaced the ‘discussion’ sub-heading with ‘conclusion’. |
| **R3Comment 3:** Authors have mentioned that "The program (NHI) has been rolled out in 75 districts, out of 76 (Health Insurance Board based on inquiry dated September 2021)". However, Nepal has 77 districts. | The authors apologize for this typo error and the information has been corrected in the revised manuscript. |
| **R3Comment 4:** Many sentences are incomplete such as "We conducted a semi-structured search using PubMed and google-scholar for reference from studies on NHI members’ retention in LMICs and Nepal published in 2015 onwards". | English editing (as mentioned in response to the R3Comment 1 above) has improved the highlighted shortcomings. |
| **R3Comment 5:** They have also mentioned that "The proposed study thus aims to achieve the following objectives in Nepal: • Analyze the factors affecting the NHI members’ intention to make annual renewals in the program • Provide concrete programmatic and policy level recommendations to strengthen the program" It seems like proposal of the study. | The authors have revised the objectives as suggested by the Editor and read as follows   - Determine the predictors of intention to renew annual subscription for NHI program among enrolled members. - Provide information to develop strategic intervention program, through policy-level recommendations, to improve renewals and strengthen the NHI program |
| **R3Comment 6:** Importantly, authors have mentioned that they have employed a concurrent mixed-method study design. The qualitative results have been published elsewhere. In my opinion, publishing findings of mixed method study separately is practice of salami publication, which is not acceptable. | The authors thank Reviewer 3 for highlighting the ethical issues related to salami publication. The authors were well aware of this and the article was submitted after careful considerations. Numerous articles, papers have defined salami as slicing referring to the practice of submitting multiple articles derived from the same data set, typically in the minimum publishable unit of research.^[1]^ ^[2]^ ^[3]^ ^[4]^ The referred papers also stated that in the absence of commonly agreed policies and guidelines on salami publication, it should be judged case by case. The authors outline the reasons why our paper does not fit into salami publication.   - The data set for the submitted article has not been analyzed and submitted to any other journal. - There are three major data sets in the larger mixed-method study; one qualitative and two quantitative. The study design, objectives, respondents, questionnaires employed to generate these data are different from each other. Please refer to Editor Comment 3 response.   Thus, the knowledge generated from these separate articles though compliment each other but offers important and unique information on its own.   - The already published paper on qualitative data is cited in the submitted manuscript. While the same published qualitative too had mentioned the quantitative data to be published elsewhere. |
| **R3 Comment 7:** The discussion section of the paper is also poorly done. Hence, I recommend rejection of this paper. | The authors are not quite sure which particular discussion sub-section is poorly written. The English editing has improved the writing flow and the discussion has been revised. Please refer to the track changed manuscript. |
| **Additional editor comments** | |
| **Editor Comment 1**: The three reviewers have considered the manuscript and made their comments for your considerations. This study is very important because of the central subject of consideration relates to access to health care, universal coverage, and burden of out-of-pocked borne by population seeking health care services in the context of public health as a population science. Importantly, factors associated with barriers to NHI enrollment have been identified from literature reviewed. | The authors extend thanks to the Editor for highlighting the study's relevance and contribution in achieving universal health coverage. We have also now highlighted this in the Discussion section. |
| **Editor Comment 2:** The abstract needs a statement of objective to indicate the course of action and direction of the study.  There is observed inadequate presentation of the methodology and results in the abstract, which needs modification. | The authors have made changes to the abstract to strengthen its content and meet the PLOS Global Health publication requirements. Please refer to the track-changed manuscript. The key changes the abstract include   - The study objective added up - The methodology is made more clearer - The result included adjusted OR values instead of p-value. - The discussion subtitle is replaced by conclusion. |
| **Editor Comment 3:** The statement describing sampling in the study would have explained how a sample of 182 participants was drawn from a larger mixed-method study. | The authors would like to state that the sample of 182 participants was independent and not drawn from a larger study. Thus, adding the suggested information might not be necessary.  The mixed-method study collected qualitative and quantitative data concurrently but from different groups of individuals. The respondents for the qualitative data were NHI policymakers, experts, program managers, field workers, and health service providers selected by the maximum variance approach. This sample was independent of the participants included in the quantitative study, whose results are presented in the present manuscript. The quantitative survey included the current NHI members and dropouts. |
| **Editor Comment 4:** There is a need to modify the study objective to read; “This study sought to determine predictors of intention to renew annual subscription for NHI program among enrolled members, provide information to develop strategic intervention programme, through policy level recommendations, to improve renewals and strengthen the NHI programme. The rest of the statements in the last sentence should be deleted. | The authors extend thanks to and Editor for this constructive suggestion to strengthen the objectives. The revised manuscript has accommodated the suggestions and the objectives as been revised. Please refer to the R3 Comment 5 response above. |
| **Editor Comment 5:** The statistical analysis and presentation of results are flawed. For instance, the tables appear busy/crowded with data. | The authors consulted with statistical experts to improve the statistical analysis and presentation. The following changes were made, as already reported above in R2Comment 3.   - The data analysis replaced chi-square with univariate logistic regression given that P-value from both processes would be similar. In particular, upon further discussion with the statistical expert, it was advised to calculate unadjusted OR (Odd Ratio) to be compared with adjusted OR, which would be only possible from univariate logistic regression. - Similarly, Pearson co-relation was employed to check the collinearity after selecting variables to include in multivariable analysis, together with VIF (Variable Inflation Factor). Since Pearson co-relation could be misleading, we omitted it from the data analysis and only retained the VIF value for clarity. - Table 2 was revised to present only descriptive statistics i.e., frequencies and percentages. - The original Table 3 was dropped. A new Table 3 would include the variables with unadjusted OR and adjusted OR with their respective p-values. |
| **Editor Comment 5**: Kindly review the comments of the reviewers carefully and make necessary modifications that would make the manuscript acceptable. | The authors would like to assure that all comments of the reviewers have been reviewed with additional support from statistical expert and the revised manuscript has incorporated all those comments. The rebuttal letter reflects all those changes in summary. |

1. Kleinert S, Wager E. Responsible research publication: international standards for editors. Pril (Makedon Akad Nauk Umet Odd Med Nauki) 2014;35:35-41.
2. Wallace MB, Bowman D, Hamilton H and Siersema PD. Ethics in publication, part 2: duplicate publishing, salami

   slicing, and large retrospective multicenter case series. 2018. The American Society for Gastrointestinal Endoscopy and Georg Thieme Verlag KG 0016-5107/$36.00

   https://doi.org/10.1016/j.gie.2018.01.002
3. Ding D, Nguyen B, Gebel K, Bauman A, and Bero L. Duplicate and salami publication: a prevalence study of journal policies

   2020. International Journal of Epidemiology, 281–288 doi: 10.1093/ije/dyz187
4. Smolčić VS Salami publication: definitions and examples. 2013. Biochemia Medica;23(3):137–41.doi.org/10.11613/BM.2013.030
